# Merging Problem-Based Learning with Simulation-Based Learning in the Medical Undergraduate Curriculum: The PAIRED Framework for Enhancing Lifelong Learning

**DOI:** 10.7759/cureus.647

**Published:** 2016-06-19

**Authors:** Jansen Koh, Adam Dubrowski

**Affiliations:** 1 The Learning Institute, The Hospital for Sick Children, University of Toronto; 2 Emergency Medicine, Pediatrics, Memorial University of Newfoundland; 3 Marine Institute, Memorial University of Newfoundland

**Keywords:** problem-based learning, lifelong learning, simulation-based learning

## Abstract

Lifelong learning is an essential trait that is expected of every physician. The CanMeds 2005 Physician Competency Framework emphasizes lifelong learning as a key competency that physicians must achieve in becoming better physicians. However, many physicians are not competent at engaging in lifelong learning. The current medical education system is deficient in preparing medical students to develop and carry out their own lifelong learning curriculum upon graduation. Despite understanding how physicians learn at work, medical students are not trained to learn while working. Similarly, although barriers to lifelong learning are known, medical students are not adequately skilled in overcoming these barriers. Learning to learn is just as important, if not more, as acquiring the skills and knowledge required of a physician. The medical undergraduate curriculum lacks a specific learning strategy to prepare medical students in becoming an adept lifelong learner. In this article, we propose a learning strategy for lifelong learning at the undergraduate level. In developing this novel strategy, we paid particular attention to two parameters. First, this strategy should be grounded on literature describing a physician’s lifelong learning process. Second, the framework for implementing this strategy must be based on existing undergraduate learning strategies to obviate the need for additional resources, learner burden, and faculty time. In this paper, we propose a Problem, Analysis, Independent Research Reporting, Experimentation Debriefing (PAIRED) framework that follows the learning process of a physician and serves to synergize the components of problem-based learning and simulation-based learning in specifically targeting the barriers to lifelong learning.

## Introduction

Lifelong learning has emerged as one of the major challenges that physicians face. Accreditation organizations, certification boards, employers, educators, and the general public view the drive for lifelong learning as an essential trait of physicians. The Canadian Medical Association Code of Ethics states that it is the responsibility of physicians to “engage in lifelong learning to maintain and improve their professional knowledge, skills, and attitudes” [1]. The CanMeds 2005 Physician Competency Framework emphasizes lifelong learning as a key competency a physician must achieve to fulfill the scholar role in becoming better physicians [2]. However, most physicians are not prepared to develop and carry out a self-directed lifelong learning plan, such as identifying their own learning needs and establishing learning goals to meet these needs. This disparity may be explained by the unfamiliarity of physicians with the lifelong learning process. They also lack the skills required to overcome the barriers to lifelong learning. Barriers to lifelong learning include unawareness of knowledge deficits, failure to formulate the right question, and inadequate researching skills, which are known to impede lifelong learning [3-4]. In addition, lack of time is often cited by the busy physician as a hindrance to learning [3]. Being competent and comfortable with developing and carrying out a self-directed lifelong learning plan could significantly reduce the task load and effort of learning, hence, minimizing time spent on learning and overcoming some of these barriers. Although the learning process of the practicing physician [5] and the barriers to lifelong learning [3-4] are well characterized in the literature, a gap exists in translating this knowledge into a learning strategy that physicians can develop for lifelong learning. The undergraduate years present an excellent opportunity to train medical students to become physicians skilled in lifelong learning. The adult learner paradox, in the context of medical education, is a phenomenon whereby adult learners revert back to the learning strategies they acquired during their undergraduate years when learning [6]. Adult learners are problem- and goal-oriented, focusing on practical solutions, and are autonomous and self-directed in their learning. Yet, when these adults are placed in a learning environment, they immediately revert back to the strategies they were exposed to and became competent in during their undergraduate years. Accordingly, equipping medical students with skills to overcome these barriers to lifelong learning and inculcating a learning strategy at the undergraduate level that is similar to the learning process of a physician may promote lifelong learning [6-7].

In this article, we propose a learning strategy for lifelong learning at the undergraduate level. In developing this novel strategy, we paid particular attention to two parameters. First, this strategy should be grounded in literature describing a physician’s lifelong learning process. Second, the framework for implementing this strategy must be based on existing undergraduate learning strategies to obviate the need for additional resources, learner burden, and faculty time. We will first examine the literature on how physicians learn at work and identify the potential barriers they face to lifelong learning. Next, we discuss the strengths and weaknesses of current undergraduate teaching/learning methods in meeting the challenges of inculcating lifelong learning skills in medical students, focusing on two of the leading educational strategies in undergraduate education, namely, problem-based learning (PBL) and simulation-based learning (SBL). Building on these two educational strategies, we propose a framework for lifelong learning that incorporates the strengths and overcomes the weaknesses of both strategies. Finally, we will explain how this framework could be implemented to effectively prepare the graduating medical practitioner for lifelong learning.

## Technical report

### PAIRED framework

We developed a framework that is built on Slotnick’s Four-Stage Theory of Physicians’ Self-Directed Learning Episodes by merging PBL and SBL for lifelong learning (Table 1) [5]. The framework is a learning strategy consisting of six sequential phases: Problem, Analysis, Independent Research, Reporting, Experimenting, and Debriefing (PAIRED). The learning exercise starts with the “Problem” phase that corresponds to Slotnick’s Stage 0: Scanning for Problems. Importantly, for PAIRED, the problem should retain the essential characteristics of that in a PBL exercise. A good PBL problem must be realistic and complex with no clear-cut answers, and one that students feel are relevant and they are likely to encounter.

**Table 1:** PAIRED Framework Build from Slotnick’s Theory Using Components of PBL and SBL PBL: Problem-Based Learning SBL: Simulation-Based Learning

Slotnick’s Theory	PAIRED Framework	PBL/SBL Components
Stage 0: Scanning for Problems	Problem	PBL and SBL
Stage 1: Evaluating the Problem	Analysis	PBL
Stage 2: Learning Skills and Knowledge	Independent Research Reporting	PBL, PBL
Stage 3: Gaining Experience	Experimentation Debriefing	SBL, SBL

However, unlike a typical paper-based PBL exercise, in PAIRED, the problem should be presented to the students through active exploration via simulated clinical encounter. Presenting the problem through simulation will better contextualize the problem for the students and mimics the way physicians identify problems in real life when working. The simulated clinical encounter not only helps students identify learning needs and gaps in knowledge and skills, it also exposes learners to the physical, emotional, environmental, and time constraints of a clinical encounter and, thus, focuses their research and reading towards more practical solutions and approaches. In fact, using a simulated clinical encounter as the clinical trigger and context for PBL has been suggested recently, albeit more research is required to assess the impact of this approach in medical education [8]. Business education literature provides evidence that simulation indeed meets the requirements of a good PBL problem [9-11]. In contrast to a typical SBL activity, the objectives of this first simulated problem step in PAIRED are strictly for problem identification and awareness of knowledge and skill deficits. These objectives should be made very clear to the students to minimize the feeling of being overwhelmed and preoccupied with diagnosis, management, and outcomes of the SBL activity at this phase. Using a simulated clinical encounter to frame and present a problem is an important first step in overcoming the barriers of being unaware of problems in a clinical setting and being oblivious to deficits in knowledge.

Next, students in the same groups as in the "Problem" phase proceed to the “Analysis” phase, where they gather in a separate room to discuss the problems that were encountered. This is very much like the initial analysis step of PBL. The facilitator should focus the students on identifying the problems encountered as well as developing their own learning needs. Facilitators should also guide students who are novice learners in formulating effective strategies to find solutions and resources to these problems. It is also important that students develop skills to address problems that are most important and relevant to their (simulated) practice. This phase is crucial for students in identifying problems and gaps in knowledge and skills because it also allows students to evaluate the problems, discuss, and decide which problems to take on. Hence, the “Analysis” phase corresponds to both Stage 0 and Stage 1 of Slotnick’s theory (Table 1). Being competent in identifying and analyzing the problem in this phase is critical for students in order to become aware of knowledge deficits and to formulate questions that are important, practical, and researchable. Students conclude this phase by deciding on how to proceed with the next phase of “Independent Research”.

The “Independent Research” phase and “Reporting” phase for the PAIRED approach are similar to the research and reporting steps of PBL. These phases also correspond to Stage 2 of Slotnick’s theory (Table 1) where students search the literature and read independently, in addition to learning through discussions with peers and experts. The “Independent Research” phase can last from under an hour to over several weeks depending on the objectives of the learning activity, the difficulty of the clinical problem, and the learning level of the students. In contrast to PBL, the “Independent Research” phase also includes learning clinical skills that learners have identified as deficiencies. It is in this phase that SBL falls short in self-directed learning as it does not incorporate any elements of independent research and reporting, other than consultation with experts and peers during debriefing. For this “Independent Research” phase to be implemented well, it is important that appropriate scaffolding, guidelines, and support are provided for students lacking experience in appraising the literature or identifying appropriate evidence from the multitude of available information. Students should be taught skills for using evidence at the point of care as this promotes learning at work [12-13] and is not a straightforward skill to attain [14-15]. As the student develops competency in identifying problems, developing learning needs, and researching skills in point of care evidence during the PAI phases of the PAIRED framework, these three initial phases can be conducted as a single session simulating clinical encounters that are embedded with specific learning opportunities that mimics everyday learning opportunities at work. Following the “Independent Research” phase, students meet again for the “Reporting” phase (as in PBL) where discussions and sharing of knowledge and skills occur, with proposed solutions to the problems that were encountered in the “Problem” phase.

The “Experimenting” phase should follow the “Reporting” phase as soon as possible so that students can proceed to test out their agreed upon and devised plans of action and management. This “Experimenting” phase follows the typical SBL activity and is the “Gaining Experience” stage of Slotnick’s theory (Table 1). The problem encountered by students in this phase should be the same problem encountered during the first “Problem” phase of the PAIRED process, allowing students to carry out their plan of action as decided in the “Reporting” phase. However, in this phase, students are expected to diagnose and manage the problems encountered. The “Experimenting” phase that follows the “Reporting” phase is crucial as the clinical outcomes simulated during the SBL exercise provide the students with immediate and contextualized feedback about the effectiveness of their “Independent Research” and “Reporting” phases. The SBL activity is an important experiential learning process that focuses students during future “Research” and “Reporting” phases on coming up with management plans that are within the practical constraints of a simulated clinical problem. This “Experimenting” phase is lacking in PBL, which is a major drawback, in our opinion, for PBL activities where students fail to make the connection between discussions and actually carrying out their solutions to see if their solutions do indeed work, thus, missing out on gaining experience through practice. The PAIRED activity finally concludes with a “Debriefing” phase, similar to the debriefing session after a typical SBL activity. The formative feedback given to students in this phase is known to be the most important factor for SBL activities in promoting effective learning [16]. This phase is where performance gaps are identified, described, investigated, and closed through discussions and targeted instructions [17]. Students will be able to learn by reflecting on their actions or inactions as well as from the performances of their peers during the debrief, an important concluding step in Slotnick’s theory that is lacking in PBL.

## Discussion

### Lifelong learning in medicine and barriers to learning

It has been established that practicing physicians are motivated to begin learning when a problem is encountered [18-19]. This problem may be specific, such as a clinical question arising from a patient encounter, or general, such as a gap in skills and knowledge due to the development of new techniques or medications. These problems encountered will then trigger the learning process that a physician moves through, from the problems that precipitate learning to the outcomes of that learning. In the Four-Stage Theory of Physicians’ Self-Directed Learning Episodes [5], Slotnick describes this learning process as four learning episodes that are based on literature characterizing practicing physicians’ learning experiences [20-22]. According to this model, the physician is aware that there will be problems at work and scans for these problems (Stage 0: Scanning for Problems). Once a problem is encountered or identified, he/she evaluates if there is a need for the problem to be addressed and evaluate the learning requirements for a solution (Stage 1: Evaluating the Problem). If he/she decides to take on the problem, learning objectives are developed, and he/she employs learning strategies to acquire the required skills and knowledge to solve the problem (Stage 2: Learning Skills & Knowledge). The physician applies the newly acquired skills and knowledge in clinical practice, gaining experience through the results and feedback from patients and colleagues (Stage 3: Gaining Experience).

Barriers to this lifelong learning process are encountered in each of these stages. To further elaborate, at Stage 0, the problem that arises during patient encounters or a realization of a gap in knowledge and skills is the starting point that motivates learning. Stage 0 is crucial and is where the physician scans for problems and identifies learning needs. Barriers to self-directed learning at this stage are the failure to identify problems that exist and a lack of awareness of knowledge deficits [4]. These are critical barriers that hamper the start of a learning process. In the words of G.K. Chesterton, *“It* *isn’t* *that* *they* *can’t* *see* *the* *solution.* *It is* *that* *they* *can’t* *see* *the* *problem.”* 

Once the physician is able to identify a problem as a learning need, he/she moves on to the next stage (Stage 1) and reflects on how relevant the problem is to her practice. The questions he/she asks herself at this stage include, “Is there a really a problem? Is a solution to the problem available? Are learning resources available to solve the problem? Is it practical for me to engage in learning?” Stage 1 is a critical stage as the learning process will be impeded if the learning is deemed futile due to unavailability of resources. Similarly, the physician may halt the learning process if the learning resources are considered extensively effortful to undertake. Being unfamiliar with the availability of resources and inability to use the resources efficiently are the obvious barriers to the learning process at this stage, and these are often referred to as a lack of time and energy [3].

Once the problem is deemed important enough to warrant a change in practice and the learning is considered practical and convenient to engage in, the physician moves on to the next stage. In Stage 2, the physician could engage in the searching and reading of relevant literature, discussing the problem and possible solutions with colleagues or experts, and taking appropriate courses. At this stage, barriers include poor researching skills in seeking out and interpreting the relevant literature, poor communication, and collaborative learning skills, as well as limited access to relevant courses. Negative experience due to failed attempts may reinforce that learning is difficult and time-consuming, and this may impede progression and limit future learning opportunities. A positive experience, on the other hand, would reiterate to the learner that self-directed learning can be efficient, useful and self-satisfying, thus, promoting lifelong learning.

Stage 2 is completed when the physician exhausts available resources, formulates a plan of action or feels that additional learning is no longer necessary. He/she will then move on to the final stage (Stage 3) of practice and gaining experience. This refers to the application and practice of the knowledge and skills acquired to resolve the perceived problem. At this stage, feedback from patients and peers on the results of the application of the newly acquired skills and knowledge are critical components of learning. The physician reflects on the feedback received and with repeated practice, integrates these skills and knowledge into everyday practice. The learning process is completed when the problem has resolved or when the physician feels that enough experience has been gained to be confident and competent in tackling similar problems in the future. Barriers to completing this stage include failure to reflect on their practice and, for conditions which are relatively uncommon, failure to gain sufficient experience due to lack of opportunities for repeated practice.

In summary, the barriers to lifelong learning include failure to identify problems, lack of awareness of knowledge deficits, poor researching skills, deficient communication and collaborative skills, limited access to resources, unfamiliarity with availability of resources, inability to use existing resources efficiently, failure to self-reflect, and lack of repeated practice (Table 2). Some of these barriers are system-based barriers, such as availability of resources and lack of opportunities for repeated practice, but most are skill-based barriers. We next examine the current learning strategies in the medical undergraduate curriculum to determine if they are suitable for overcoming skill-based barriers to lifelong learning.

**Table 2:** Targeting Barriers to Lifelong Learning *See text for further details

Four-Stage Theory of Physicians’ Self-Directed Learning Episodes	Barriers to Learning	Do these Learning Strategies Target Barriers to Learning?		
		PBL	SBL	PAIRED
Stage 0: Scanning for Problems	Failure to identify problem	Partial*	Yes	Yes
	Lack of awareness of knowledge deficit	Partial*	Yes	Yes
Stage 1: Evaluating the Problem	Unfamiliar with availability of resources	Yes	No	Yes
	Inefficient use of resources	Yes	No	Yes
Stage 2: Learning Skills and Knowledge	Poor researching skills	Yes	No	Yes
	Poor collaborative skills	Partial*	Yes	Yes
Stage 3: Gaining Experience	Failure of self-reflection	Partial*	Yes	Yes
	Lack of repeated practice	No	Yes	Yes

### Strategies for lifelong learning

The undergraduate medical curriculum offers a crucial window period for students to acquire a lifelong learning strategy and overcome skill- based barriers to lifelong learning*.* The tendency for physicians to adopt learning strategies gained during their undergraduate years, i.e. adult learner paradox, underscores the importance of an effective lifelong learning strategy at the undergraduate level. The undergraduate medical curriculum consists of a variety of teaching/learning strategies, such as lectures, clinical tutorials, mentoring, PBL, and SBL. While “traditional” teaching methods, such as lectures, are important in the medical undergraduate curriculum in providing knowledge and information in an economical and resource efficient way [23-24], both PBL and SBL are increasingly used worldwide in medical schools to promote independent thinking, self-directed learning, effective collaboration, and application of knowledge [25]. PBL and SBL can potentially address some of the shortcomings of traditional curricula in providing lifelong learning skills. Hence, we will focus on how these strategies promote lifelong learning for the practicing physician.

(i) Problem-Based Learning (PBL):

PBL is a learning strategy for “posing contextualized, real world situations and providing resources, instruction and guidance to learners as they develop content knowledge and problem-solving skills” [26]. Burch described PBL as a process involving four sequential steps: the problem, initial analysis, research, and reporting [27]. The PBL process typically starts with a paper-based problem that is complex and authentic. Next, in the initial analysis step, students gather in small groups to identify specific learning issues. By inventorying what is known from their prior knowledge and personal experience, students are made aware of their gaps in knowledge and skills. The research step refers to the independent self-directed learning engaged in by the student. This can be at the library, via the internet or by interviewing authoritative sources. Finally, the reporting step concludes the PBL exercise where students gather again to report their discoveries, explain concepts, clarify doubts, and field questions from members.

PBL has several strengths in fulfilling the theory of lifelong learning of physicians when implemented properly. However, as shown in Table 2, PBL falls short in certain aspects. A PBL exercise typically starts with a paper-based clinical problem where students gather together in small groups to identify problems, learning needs and knowledge gaps. This only partially satisfies Stage 0 because, in reality, the physician must be able to achieve these learning goals through interaction with a patient or from a clinical experience, which is evidently different from a paper-based clinical scenario [28]. We feel that it is important that medical students develop the skills to overcome barriers at Stage 0 through more realistic interactions rather than through a paper-based problem.

Furthermore, PBL only partially addresses the barrier of collaborative learning. While PBL has been shown to improve communication skills of physicians [29], it does not adequately prepare the student to learn while working with colleagues during clinical encounters. Having a discussion to come up with a solution is very different from collaborative learning work. While physicians can learn from peers through discussions about the case, learning on the job from colleagues involves not only discussions but also observations of behavior, communications, and skills that their colleagues demonstrate while interacting with a patient or dealing with a clinical situation [30]. Physicians often learn through picking up “tricks of the trade” and clinical pearls from their colleagues that are not explicitly discussed at work.

The lack of practice and opportunity to reflect on one’s performance in a clinical encounter is a significant drawback of PBL concerning the theory of physicians’ learning. The PBL exercise typically ends with a small group discussion on the solutions to the case as well as in-depth discussion of the problems. However, students do not get a chance to practice and “act out” their solutions and approaches which are crucial. By practicing a devised approach, learners will have the opportunity to realize the many constraints that they may not be aware of. This realization will sharpen their researching skills and focus discussions towards workable solutions. Gaining experience occurs when newly acquired knowledge and skills are applied and practiced. This is the final stage of a physician’s learning process.

(ii) Simulation-Based Learning (SBL):

SBL is defined by Gaba as “a technique to replace or amplify real experiences with guided experiences, often immersive in nature, that evoke or replicate substantial aspects of the real world in a fully interactive manner” [31]. The SBL exercise is a small group learning activity that typically starts with a pre-briefing and orientation to the simulation room, the simulator, and all other equipment used during the simulation. A simulated problem is then presented to the students who actively participate in resolving the problem, working with one another as a team. Clinical responses and results simulated during the exercise serve as feedback to students on the consequences of their actions or inactions. The SBL activity concludes with a debriefing session, which is crucial to learn.

The major shortcoming of SBL with respect to the physicians’ learning process is the lack of time and skills training devoted to the crucial components for self-directed learning. Specifically, familiarity with learning resources, efficient use of learning resources, and researching skills are not addressed with SBL (Table 2). Performing a comprehensive search and identifying the most appropriate up-to-date evidence to solve problems identified requires training and practice [6]. Similarly, filtering out non-relevant information is just as important during the learning process. These important skills are not addressed with SBL in medicine. While SBL has characteristics that target several barriers to lifelong learning, failure to address barriers at Stage 1 and 2 would halt the self-directed learning process.

(iii) Integrating PBL and SBL for Synergism: 

Both PBL and SBL have the components essential for inculcating lifelong skills in medical undergraduate students. In fact, PBL and SBL complement each other to fulfill the theory of physicians’ lifelong learning and overcome skill-based barriers to lifelong learning (Table 2). However, is it enough to have these learning strategies as separate educational tools in the undergraduate curriculum for lifelong learning to occur? Could additional benefit be derived from integrating SBL and PBL in a coordinated strategy so that the complementary strengths of one can fill the gap left by the other? We propose that PBL and SBL should be integrated specifically to develop lifelong learning as based on Miller’s pyramid of performance assessment, the importance of deliberate practice, and the value of curriculum integration.

**Figure 1:**
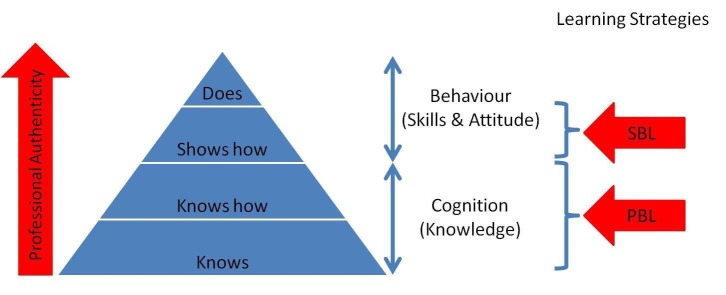
Mapping SBL and PBL to Miller's Pyramid of Clinical Competence

Miller’s pyramid is used as a framework for the assessment of clinical competence and as a tool for the development of assessment methods [32]. However, it may also be viewed as a framework for formative assessment and the development of learning activities. In medical education, PBL targets the “knows” and “knows how” levels whereas SBL targets the “shows how” levels (Figure 1). By integrating PBL with SBL for lifelong learning activities, students will experience increasing professional authenticity and apply the knowledge they have acquired from PBL through SBL. Learning activities should include both cognition (PBL) and behavior (SBL) levels in Miller’s pyramid. These are the knowledge, skills, and attitudes that students should acquire by merging PBL with SBL for lifelong learning. From the perspective of formative assessment, Miller’s pyramid argues for an integrated learning strategy in providing a holistic education in lifelong learning.

Deliberate practice refers to the repetitive practice of cognitive or psychomotor skills with the goal of improved performance and development of expertise through specific feedback and rigorous skills assessment [33]. The Four-Stage Theory of Physicians’ Self-Directed Learning Episodes should be viewed as a skill set that medical students should acquire for lifelong learning. Hence, students should engage in the deliberate practice of this four-step learning process to overcome the skill-based barriers in self-directed learning to become experts in lifelong learning. Accordingly, PBL and SBL should be integrated into a learning strategy that follows the Four-Stage Theory of Physicians’ Self-Directed Learning Episodes*.* This integrated learning strategy needs to be practiced repeatedly in various simulated clinical encounters in order to achieve expertise in self-directed learning upon graduation and promote lifelong learning as a key competency. Hence, having PBL and SBL as separate educational strategies may not be well-suited for developing the required competency in lifelong learning.

Curriculum integration is a key principle for sound SBL in medical education. In a study published by McGaghie, et al. [8], the authors stated that SBL should be “carefully integrated with other education events, including… PBL…”. Curriculum integration results in content integrity and enhances authenticity, enabling students to develop a unified view of the curriculum that reflects the real world. Students who engage in an integrated learning strategy of PBL and SBL will become skilled in the entire learning process of a physician and achieve an understanding of learning while working. Having to integrate the skills obtained from PBL and SBL on their own may be an impediment to lifelong learning. It is our hope that by instilling a learning strategy that follows the self-directed learning process of the practicing physician, it would promote lifelong learning in becoming better physicians, a goal of undergraduate curriculum [2].

Having both PBL and SBL as separate teaching strategies in the undergraduate in the curriculum is not sufficient to meet lifelong learning needs of medical students. To achieve this goal, we propose to synergize the two existing educational strategies, PBL and SBL, via a framework that follows the learning process of a physician and targets the skill-based barriers to lifelong learning.

## Conclusions

Lifelong learning skills are expected of every physician and, therefore, should be addressed at the undergraduate level. Translating knowledge of how physicians learn and the barriers they face into a learning strategy for undergraduate medical students is important in preparing them for lifelong learning. Skill-based barriers to lifelong learning could be overcome with the right training and educational strategy. The PAIRED framework for implementing a novel strategy for lifelong learning closely follows the learning process of physicians and utilizes the components and resources of current undergraduate learning strategies in targeting these barriers to promote lifelong learning competency. The PAIRED framework serves to synergize both PBL and SBL to achieve the objective of acquiring lifelong learning competency. Students who become competent in the PAIRED framework in their undergraduate years may find the lifelong learning process of a physician more intuitive. Merging PBL and SBL via the PAIRED framework may also enable students to better appreciate learning in medicine through curriculum integration. By engaging in the deliberate practice of the PAIRED framework, it is our hope that students will develop lifelong learning competencies.
